# Mitochondrial dysfunction and neurodegeneration in multiple sclerosis

**DOI:** 10.3389/fphys.2013.00169

**Published:** 2013-07-25

**Authors:** Kimmy Su, Dennis Bourdette, Michael Forte

**Affiliations:** ^1^Vollum Institute, Oregon Health and Science UniversityPortland, OR, USA; ^2^Department of Neurology, Oregon Health and Science UniversityPortland, OR, USA; ^3^Department of Veterns Affairs, VA MS Center of Excellence-WestPortland, OR, USA

**Keywords:** mitochondria, p66ShcA, neuronal viability, neurodegeneration, multiple sclerosis

## Abstract

Multiple sclerosis (MS) has traditionally been considered an autoimmune inflammatory disorder leading to demyelination and clinical debilitation as evidenced by our current standard anti-inflammatory and immunosuppressive treatment regimens. While these approaches do control the frequency of clinical relapses, they do not prevent the progressive functional decline that plagues many people with MS. Many avenues of research indicate that a neurodegenerative process may also play a significant role in MS from the early stages of disease, and one of the current hypotheses identifies mitochondrial dysfunction as a key contributing mechanism. We have hypothesized that pathological permeability transition pore (PTP) opening mediated by reactive oxygen species (ROS) and calcium dysregulation is central to mitochondrial dysfunction and neurodegeneration in MS. This focused review highlights recent evidence supporting this hypothesis, with particular emphasis on our *in vitro* and *in vivo* work with the mitochondria-targeted redox enzyme p66ShcA.

## Multiple sclerosis as a neurodegenerative disorder

**Multiple sclerosis** (MS) is one of the most common neurological diseases plaguing more than 2.1 million people worldwide. About 85% of patients present with a relapsing-remitting phenotype during which they experience clinical attacks, or relapses, causing neurological dysfunction including optic neuritis and transverse myelitis. About 50% of these patients eventually transition into a secondary progressive phenotype characterized by continuously worsening neurological function leading to significant permanent disability. About 15% of patients have disease that is progressive from onset, referred to as primary progressive MS (Noseworthy et al., 2000).

Traditionally, MS has been considered an autoimmune disorder in which T cells, macrophages, soluble mediators of inflammation, and autoantibodies contribute to multifocal demyelination. Currently available treatment options, which include interferon-beta, glatiramer acetate, natalizumab, and fingolimod, are designed to control the pathogenic inflammatory processes through varying mechanisms. While these medications have proven to be beneficial in limiting the frequency of acute relapses in MS patients, they are minimally effective in preventing the progressive neurological decline that contributes largely to long-term disability in people with MS. Consequently, it is now clear that the disease is much more complex and not purely an autoimmune disease driven by the adaptive arm of the immune system. Besides the appearance of episodic focal inflammation lesions, there is a more generalized and progressive disease process that results in slow axonal and neuronal degeneration. The pathogenesis of this neurodegenerative process is uncertain. It may be driven by release of ROS by activated microglia or by axoplasmic accumulation of Ca^2+^ resulting for ionic imbalances within chronically demyelinated axons. Therefore, it is likely that other mechanisms are involved in MS disease pathophysiology and hence need to be addressed.

Of note, the famous neurologist Jean Martin Charcot mentioned in his early descriptions of MS that axonal pathology may play a role in the disease. However, given technological limitations, it was not until more recently with the development of advanced *in vivo* and *in vitro* imaging techniques that there was solid evidence that **neurodegeneration** occurs early in both gray and white matter of MS patients (Lovas et al., 2000; Kuhlmann et al., 2002; Ingle et al., 2003; Kutzelnigg et al., 2005; Herrero-Herranz et al., 2008). For example, immunohistochemistry, confocal imaging, and 3-D computer reconstruction of post-mortem MS patient brains showed a significant amount of transected axons in both active and chronic active white matter lesions (Trapp et al., 1998). In addition, axonal degeneration analysis in chronic inactive lesions of secondary progressive MS patient spinal cords using similar methods indicated a 61% reduction in axonal density (Bjartmar et al., 2003). Likewise, analysis of normal appearing white matter (NAWM) showed decreased nerve fiber densities in patient spinal cords (Bjartmar et al., 2001). These postmortem results have been confirmed by *in vivo* studies utilizing magnetic resonance spectrometry (MRS), which determines the chemical composition of brain matter via analysis of certain peaks in the resonance spectra. In particular, the metabolite N-acetyaspartate (NAA) produced by neuronal mitochondria is considered an important monitor of neuronal integrity, and decreases in NAA have been correlated with relapses and neurological decline in MS patients (Fu et al., 1998; Signoretti et al., 2001; Lu et al., 2004; Benarroch, 2008). Furthermore, NAA levels are decreased in MS white matter lesions and NAWM as well as gray matter, suggesting that neurodegenerative processes are widespread throughout the central nervous system (CNS). Interestingly, gray matter atrophy rates have been correlated with worsening disability as shown by a 3.4-fold increase in atrophy from clinically isolated syndromes to relapsing remitting MS, and a 14-fold increase in atrophy from relapsing-remitting to secondary progressive MS. In contrast, white matter atrophy rates were constant at 3-fold across the MS stages (Fisher et al., 2008).

## Mitochondrial dysfunction and neurodegeneration in MS

One of the mechanisms hypothesized to explain the diffuse neurodegeneration found in MS patients involves **mitochondrial dysfunction**. Mitochondria are essential intracellular organelles involved in ATP synthesis and calcium regulation and are also major sources of **reactive oxygen species** (ROS). Therefore, diffuse mitochondrial dysfunction secondary to MS disease processes could lead to inadequate energy production and intracellular dysregulation. This dysfunction is particularly damaging to neurons given their unique elongated morphology and dependence on ATP to propagate electrical signals, maintain ionic gradients, and facilitate anterograde and retrograde transportation along axons.

There is accumulating *in vitro* and *in vivo* evidence of mitochondrial dysfunction in MS. For one, NAA, the commonly used MRS marker of neuronal integrity, is produced by neuronal mitochondria. Therefore, changes in NAA levels can also reflect mitochondrial dysfunction within neurons (Fu et al., 1998; Signoretti et al., 2001; Benarroch, 2008). As previously mentioned, changes in NAA levels correspond with relapses and have been shown to fall dramatically in acute inflammatory lesions and partially reverse as inflammation subsides (Fu et al., 1998; Khan et al., 2005). The initial dramatic decline in NAA undoubtedly reflects reversible mitochondrial dysfunction in neurons within these acute lesions. Similar NAA decreases in chronic focal white matter lesions and NAWM also may suggest chronic mitochondrial dysfunction within neurons as well as neuronal loss (Bjartmar et al., 2000). In addition, evidence linking mitochondrial dysfunction to neurodegeneration has been shown by post-mortem analysis of MS patient brain tissue, including lesioned areas, NAWM, and nonlesional cortex. Structurally, electron microscopy of demyelinated spinal cord lesions show dramatically decreased numbers of mitochondria and microtubules (Dutta et al., 2006). Further analysis indicates oxidative damage of mitochondrial DNA and impaired activity of mitochondrial enzyme complexes in lesioned tissue (Lu et al., 2000), and reduced mitochondrial gene expression specific to neurons in nonlesional tissue (Dutta et al., 2006). Finally, there is compelling evidence of mitochondrial dysfunction from *in vivo* imaging of double-transgenic mice with CFP-labeled mitochondria and YFP-labeled axoplasm (Thy1-MitoCFP × Thy1-YFP-16) induced with experimental autoimmune encephalomyelitis (EAE), a commonly used animal model for MS (Nikic et al., 2011). In monitoring single axons in the EAE-induced animals, abnormal mitochondrial morphology was noted to be the earliest ultrastructural sign of damage, preceding structural changes. Subsequent analysis of active lesions in MS patient biopsies showed similar mitochondrial damage in both structurally intact and demyelinating axons. Overall, these multifaceted studies provide convincing evidence that mitochondrial function may play a significant role in MS neurodegenerative mechanisms.

## Mitochondrial dysfunction and the permeability transition pore (PTP)

We have proposed that mitochondrial dysfunction in MS may be mediated by the pathological opening of the **mitochondrial permeability transition pore** (PTP). The PTP is a transient pore located in the inner mitochondrial membrane that when open allows solutes with molecular masses up to 1500 Daltons to enter. The pore transiently opens to certain stimuli, including calcium, reactive oxygen and nitrogen species. Persistent pathological pore opening induces the influx of solutes into the mitochondrial matrix resulting in loss of the mitochondrial membrane potential and equilibrium of ionic gradients, which can prevent ATP synthesis, and promote matrix expansion, mitochondrial swelling, and membrane rupture leading to release of cytochrome c into the cytoplasm and eventual cell death (Bernardi et al., 2006).

## Cyclophilin D and the PTP

Cyclophilin D (CyPD), a mitochondrial matrix protein, plays a crucial regulatory role in PTP opening. Various pharmacologic and genetic techniques have been used to alter CyPD activity and expression, including cyclosporin A (CsA) administration, which inhibits CyPD, and deletion of the murine *Ppif* gene, which encodes CyPD. CsA treatment has long been shown to inhibit PTP opening, and has been tested in several *ex vivo* and *in vivo* models of disease, including ischemic-reperfusion injury of the heart, and ischemic and traumatic brain injury (Griffiths and Halestrap, 1993; Friberg et al., 1998). In particular, CsA pre-treatment of insulin-induced hypoglycemic mice has been shown to significantly reduce brain damage, and ultrastructural examination suggests that the marked protection is due in part to mitochondrial preservation. Alteration of CyPD activity by *Ppif* gene deletion (CyPD-knockout; CyPD-KO) has resulted in viable animals that still can form the PTP but have a higher mitochondrial Ca^2+^ set point for PTP activation. Consequently, the mitochondria in CyPD-KO mice are able to retain about double the amount of Ca^2+^ as mitochondria from wild-type (WT) animals before PTP activation, demonstrating that CyPD is a significant regulator of PTP opening (Baines et al., 2005; Basso et al., 2005).

Due to the altered PTP properties in CyPD-KO mice, they have been used in studies addressing mitochondria dysfunction and disease pathology. For instance, CyPD-KO mice subjected to cardiac ischemia/reperfusion showed a 40% reduction in myocardial infarction compared to WT mice (Baines et al., 2005). Similarly, Schinzel et al. found that CyPD-KO mice were significantly protected from brain ischemia/reperfusion injury via middle cerebral artery occlusion, demonstrating a 62% reduction in brain infarct size (Schinzel et al., 2005). These findings suggest that CyPD elimination and ensuing alteration of PTP properties generates significant protection in various disease models and organ systems.

To address the mitochondrial hypothesis of neurodegeneration in MS, CyPD-KO mice were induced with EAE (Forte et al., 2007). EAE is a well-recognized animal model for MS, and involves immunization with fragments of myelin proteins, including myelin oligodendrocyte glycoprotein (MOG) and proteolipoprotein (PLP). In this study, C57BL/6 background CyPD-KO and WT mice were immunized with MOG 35-55 peptide to induce EAE. The immunized CyPD-KO mice developed clinical signs of hindlimb and forelimb weakness and paralysis, but unlike the WT mice, partially recovered. Histologic analysis of CyPD-KO spinal cords showed significantly decreased axonal damage and loss compared to WT cords despite having similar levels of CD4+ T cells and CD11b+ macrophages/microglia. Furthermore, isolated CyPD-KO neurons were more resistant to treatment with H_2_O_2_ and NO, and CyPD-KO brain mitochondria were able to sequester substantially greater amounts of Ca^2+^. These results indicate that regulation of PTP opening and mitochondria integrity has a significant effect on EAE disease progression and axonal survival.

## Reactive oxygen species and the PTP

Further study of mitochondrial dysfunction in the context of the PTP has led to the investigation of critical upstream promoters of PTP opening, including ROS (Bernardi et al., 2006). In terms of ROS, these are potent intracellular oxidants that include hydrogen peroxide, superoxide, and hydroxyl radicals. They are commonly produced by dedicated enzymes, the NADPH oxidases, and as metabolic byproducts due to electron leaks by the electron transport chain (Esposito et al., 1999; Turrens, 2003; Bedard et al., 2007). Because of their potential damaging effects on cellular macromolecules such as nucleic acids, proteins, and lipids, ROS levels are tightly regulated by intracellular superoxide dismutases and catalases. However, various stressors can dramatically increase ROS levels leading to significant intracellular damage. This is especially true for mitochondria, which are major sites of ROS production due to aerobic respiration and oxidative phosphorylation.

Recently, yet another pathway for mitochondrial ROS production has been discovered involving the **p66ShcA** (p66) protein (Giorgio et al., 2005; Hajnóczky and Hoek, 2007; Pinton et al., 2007). The *ShcA* gene encodes two mRNA species, one specific for p66 and one, by the use of alternate translation start sites, encoding the p46 and p52 isoforms (Migliaccio et al., 1999). While all three isoforms share the same amino acid sequence at the C terminus, including phosphotyrosine-binding (PTB), Src homology 2 (SH2), and collagen homology (CH1) domains, only the p66 isoform has a unique N terminus that allows it to be serine phosphorylated and targeted to mitochondria. Consequently, in contrast to p46 and p52, p66 is not involved in the transmission of activation signals from receptor tyrosine kinases to Ras proteins (Migliaccio et al., 1997). Rather, the p66 isoform gets serine phosphorylated by protein kinase C-Beta (PKC-β) in the cytoplasm, and following interaction with isomerase Pin1, subsequently targets to the mitochondrial intermembrane space. There, it serves as a redox enzyme by oxidizing cytochrome c, and transferring electrons to reduce oxygen into superoxide and hydrogen peroxide (Giorgio et al., 2005). This localized pool of mitochondrial ROS is proposed to mediate PTP opening and cell death activation.

**Figure 1:**
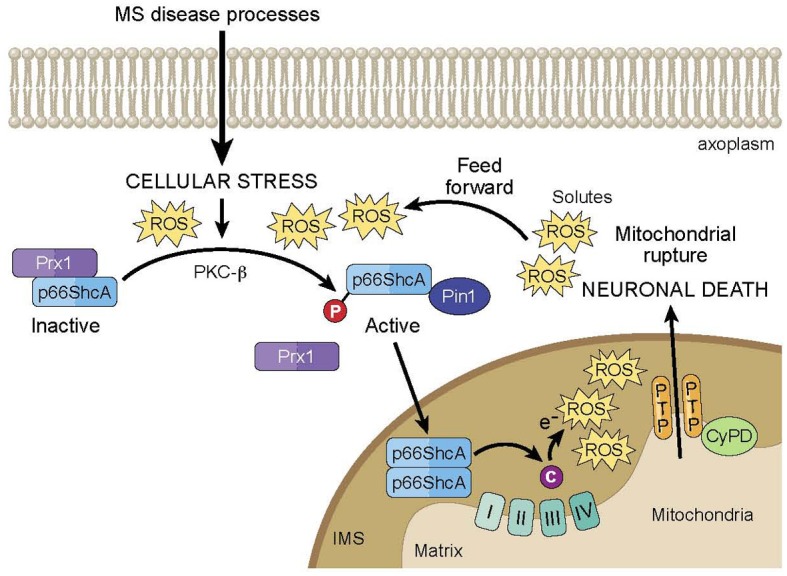
**Model of p66ShcA-mediated feed forward pathway of mitochondrial ROS and neurodegeneration in MS**. Under normal cellular conditions, p66ShcA is sequestered by Prx1 in an inactive form. Under the excessive cellular stressors associated with MS disease processes, p66ShcA is disassociated from Prx1 and is serine phosphorylated by PKC-β. Phosphorylated p66ShcA forms a complex with Pin1 isomerase, which leads to p66ShcA translocation into the intermitochondrial space (IMS). There, p66ShcA oxidizes cytochrome c and reduces oxygen to form mitochondrial ROS, which induces opening of the mitochondrial PTP. PTP opening subsequently induces the influx of solutes into the mitochondrial matrix resulting in loss of mitochondrial membrane potential and equilibrium of ionic gradients, which can prevent ATP synthesis, and promote matrix expansion, mitochondrial swelling, and membrane rupture leading to release of cytochrome c into the axoplasm and eventual neuronal death. Importantly, p66ShcA is part of a positive feed-forward signaling pathway that further elevates oxidative stress levels and activates mitochondria-mediated neuronal death.

Furthermore, p66 is part of a positive feed-forward signaling pathway for mitochondria-mediated cell death, serving as both a ROS sensor and amplifier (Figure 1). At typical cellular conditions, cytoplasmic p66 is unphosphorylated, and according to various studies in mitochondrial preparations, is sequestered in a ROS stress-sensing complex that keeps p66 inactive at low to moderate stress levels. In particular, peroxiredoxin (Prx) 1 has been proposed to be part of this complex (Gertz et al., 2009). Prxs are a family of thiol-based H_2_O_2_-degrading enzymes, so-called peroxidases, and are weak scavengers with the ability to degrade low levels of cellular H_2_O_2_. At these conditions, the stress-sensing complex consists of Prx1 in its dimerized, active form, and p66 in its dimerized, inactive form. However, at elevated levels of cellular stress, Prx1 function becomes overwhelmed, and the Prx1/p66 complex disassembles, freeing up p66 for activation. In addition, it has been shown that upon induction by stress factors, p66 expression increases and existing p66 becomes stabilized, further amplifying the pool of cytosolic p66 available for serine phosphorylation by stress activated PKC-β. Phosphorylated p66 translocates into the mitochondrial intermembrane space following interaction with Pin1 isomerase, and there, it serves as a ROS amplifier by generating mitochondrial ROS to induce PTP opening, which further elevates oxidative stress levels and activates mitochondria-mediated cell death.

Efforts to analyze the potential protective effects of p66 elimination have led to *in vitro* and *in vivo* studies of p66 elimination by genetic manipulation. For instance, p66-knockout (p66-KO) mouse embryonic fibroblasts (MEFs) were found to have increased resistance to H_2_O_2_ treatment compared to WT MEFs, and notably, this greater viability could be significantly reduced via retroviral-induced p66 expression (Migliaccio et al., 1999). Furthermore, assessment of mitochondria morphology in the stressed MEFs showed that p66-KO MEF mitochondria remained intact and elongated following H_2_O_2_ treatment compared to the noticeably swollen and fragmented WT MEF mitochondria (Pinton et al., 2007). In the *in vivo* setting, p66-KO mice were also more resistant to oxidative stress as indicated by their response to paraquat treatment, which induces superoxide production upon cellular intake. Treated p66-KO mice had a 40% increase in mean survival time compared to WT mice (Migliaccio et al., 1999). It is likely due to such protective effects that p66-KO mice have significantly prolonged lifespans, around 30% longer than those of WT mice, leading to the idea that p66 is a longevity gene. However, more recent studies have indicated that the longer life span of the mice can only be observed in protected laboratory conditions (Giorgio et al., 2012).

In the context of human disease, p66 elimination has been shown to be protective in various disease models and organ systems. In particular, p66-KO mice maintained on a chronic 21% high fat diet had significantly decreased aortic cumulative early lesion area compared to WT mice (21% in WT; 3% in p66-KO), and immunohistochemical analysis showed less macrophage-derived foam cells and apoptotic vascular cells in the p66-KO lesions (Napoli et al., 2003). Similarly, p66-KO mice used in a diabetes disease model demonstrated less marked changes in renal structure and function as evidenced by significantly lower levels of proteinuria, albuminuria, glomerular sclerosis index, and glomerular and mesangial areas, as well as decreased apoptotic cell death (Menini et al., 2006). Recent studies have shown however that in response to the elimination of p66 in this KO line, another Shc isoform, p46 has a fourfold increased expression in white fat (Tamilov et al., 2011). As a result, it has been suggested the p46 overexpression in white fat, rather than p66-KO, is might be the cause of decreased adiposity and reduced insulin-sensitivity in the fat of p66-KO mice. The protective effects of p66 also extended to other diseases including ethanol-induced liver damage, in which p66-KO mice fed with an alcohol-rich diet showed reduced liver swelling, serum ALT levels and attenuated fatty changes compared to WT controls (Koch et al., 2008).

We utilized these p66-KO mice to investigate whether altering mitochondrial ROS production might be neuroprotective in the context of EAE (Su et al., 2012a). Similar to the aforementioned experiments on CyPD-KO mice, the p66-KO mice were induced with EAE by MOG 35-55 peptide immunization and monitored for clinical signs of hindlimb and forelimb weakness and paralysis over a 36-day period. Overall, clinical protection was observed in the p66-KO mice compared to WT mice following EAE induction. Throughout the entire observation period, the p66-KO mice had consistently lower mean EAE scores compared to the WT mice. Furthermore, the p66-KO mice had significantly lower mean EAE scores at the end of the observation period, reflecting the consistent trend of reduced clinical impairment with p66 elimination.

Following the 36-day observation period, spinal cords and optic nerves were processed for histological analysis via plastic embedding and toluidine blue staining. Sections were manually quantified for pathological changes, including swollen axons, degenerating axons, demyelinated axons, irregularly myelinated axons, myelin oviods, microcysts, and areas devoid of axons. Results indicated significant protection in both optic nerves and spinal cord sections of the p66-KO mice with EAE compared to WT controls, roughly 38 and 42% decreases in neuronal pathology, respectively. Furthermore, immunohistochemical analysis of immune cell infiltration within the p66-KO and WT tissue showed that the neuroprotection observed with p66 elimination was not attributable to a reduced inflammatory response following EAE induction. Specifically, CD4 and CD11b staining for T cells and macrophages were similar between the p66-KO and WT mice with EAE. Similar responses were also noted for proliferation assays and cytokine profiling with MOG 35-55 peptide stimulation. Overall, these results suggest that the neuroprotective effects of p66 elimination were not attributable to altered MOG 35-55 peptide immunoreactivity and reduced CNS inflammation with EAE.

We next investigated the neuroprotective effects of p66 elimination at an *in vitro*, sub-cellular level by manipulating hippocampal neuronal cultures of p66-KO and WT mice (Su et al., 2012b). Briefly, hippocampal cultures were treated with varying concentrations of H_2_O_2_ and nitric oxide donor diethylenetriamine/nitric oxide adduct (DETA-NO) and assessed for neuronal viability and changes in mitochondrial structure and function. As expected, greater cell viability was demonstrated in the p66-KO cultures compared to WT cultures at the various treatment concentrations of H_2_O_2_ and DETA-NO. In terms of mitochondrial structure and function, changes in mitochondrial morphology and mitochondria-localized ROS were examined. Previous studies have shown that morphology changes correlate with eventual neuronal damage under various noxious conditions associated with neurodegenerative diseases (Solenski et al., 2002; Nikic et al., 2011). Mitochondria, which are normally thin and elongated in morphology, become swollen and fragmented prior to notable structural damage of axons, and therefore may serve as a key marker of neuronal distress. Furthermore, increased intracellular production of oxidative agents, in particular, mitochondria-localized ROS, has been shown to exacerbate neuronal distress in multiple neurodegenerative disease models (Fiskum et al., 2003; Scherz-Shouval and Elazar, 2007). The results from the p66-KO and WT studies demonstrated that mitochondrial morphology changes were less prominent in the p66-KO neurons compared to the WT neurons, with significantly greater preservation of mitochondrial length following treatment with either H_2_O_2_ or DETA-NO. In addition, the p66-KO neurons exhibited less elevated mitochondrial ROS levels compared to WT neurons following oxidative insults. Overall, the results suggest that p66 elimination incurs greater neuronal robustness and preservation of mitochondrial integrity following oxidative insults implicated in EAE- and MS-associated degenerative mechanisms.

Importantly, these findings support our current understanding of p66 and its role in cellular responses to oxidative challenges. As outlined earlier, p66 is thought to serve as a ROS sensor and amplifier under conditions of cellular stress by amplifying ROS generation via cytochrome c oxidation and oxygen reduction (Giorgio et al., 2005). Subsequently, elevated ROS has been shown to induce PTP opening as demonstrated by mitochondrial swelling, rupture, and release of cytochrome c to activate apoptotic pathways (Petronilli et al., 1994; Vercesi et al., 1997; Yang et al., 2007). The results reported here are consistent with this proposed pathway, as shown by the higher ROS levels in WT neurons compared to p66-KO neurons following stresses induced by H_2_O_2_ or DETA-NO treatment, demonstrating that p66 elimination reduces ROS amplification following these insults. This, in turn, provides greater preservation of mitochondrial integrity as visualized by mitochondrial morphology. Previous studies have correlated changes in mitochondrial morphology with mitochondrial integrity and activity, including the balance of fission and fusion events and mitophagy of damaged mitochondria. In particular, it has been shown that the dissipation of the mitochondrial membrane potential affects fusion and induces mitochondrial fragmentation (Legros et al., 2002). Furthermore, mitochondrial morphology changes may be correlated to matrix swelling mediated by pathologic PTP opening, release of cytochrome c, and activation of cell death pathways (Scorrano et al., 2002). Assuming that p66 elimination alters the cell crisis signal of elevated mitochondrial ROS, preserves mitochondrial integrity, and subsequently regulates PTP-mediated cell death, this would suggest greater neuronal robustness in p66-KO neurons following these stresses, as is supported by the cell viability studies.

## Indications for mitochondria-targeted neuroprotective treatments

Overall, the characterization of p66 elimination in neurons supports previous studies demonstrating mitochondrial integrity as a key marker of neuronal viability and fate (Nikic et al., 2011). Hence, therapeutics promoting mitochondrial preservation may prove essential to current treatment regimens for neurodegenerative diseases including MS. Mitochondria-directed therapies are still in their infancy, largely limited to *in vitro* and *in vivo* animal studies to test their properties and efficacy, though a few are now being investigated in human clinical trials. They span a wide range of mitochondria-specific targets including the electron transport chain, ATP synthase, mitochondrial ROS, and the PTP (Camara et al., 2009). Indeed, recent findings indicating that the PTP is formed by dimers of ATP synthase will suggest that this enzyme complex may also function as a molecular switch that signal activation of cell death pathways. The detailed mechanism through which this transition is achieved are not the subject of intense study on a number of levels and will refocus the search for pharmacological modifier of the activity of the PTP. In terms of drugs targeting mitochondrial ROS, these include mitochondria-directed antioxidants such as MitoQ and MitoVit E. Both compounds utilize lipophilic agent TPP+ to increase uptake into mitochondria, which has been shown to be more effective than untargeted antioxidants in combating oxidative stress (Smith et al., 1999; Adlam et al., 2005). Another unique class of mitochondria-targeting compounds includes the Szeto-Schiller (SS) peptides, which have an aromatic-cationic motif that allows them to be cell permeable and to selectively target the intermitochondrial membrane. Unlike MitoQ and MitoVit E, the SS peptides do not require mitochondrial membrane potential for uptake, which may be beneficial when dealing with diseased mitochondria with reduced mitochondrial membrane potentials (Szeto, 2008). One of the more studied SS peptides, SS-31, has been shown to scavenge matrix H_2_O_2_ and ONOO^−^, inhibit lipid peroxidation, reduce mitochondrial swelling, and reduce cytochrome c release, with notable protective effects in an amyotrophic lateral sclerosis (ALS) animal model as well as the MPTP model of Parkinson's disease (Petri et al., 2006; Yang et al., 2009). Besides targeting mitochondrial ROS, another potential mitochondria-specific target is the PTP, in particular the PTP regulator CyPD. CyPD inhibitor CsA and its non-immunosuppressant derivatives have been shown to provide significant protection for cultured neurons induced with hypoglycemic, oxidative, and ischemic damage, as well as in several kidney, liver, and heart disease models (Camara et al., 2009). As for novel therapeutic targets for mitochondrial preservation, the recent work on p66 suggests that pharmacologic inhibition of this ROS sensor and amplifier may provide neuroprotective benefits in the context of MS and perhaps other neurodegenerative diseases as well. Finally, recent advances indicating that the PTP is formed by dimers of ATP synthase suggest that this enzyme complex may also function as a molecular switch that signals activation of cell death pathways (Bernardi, 2013; Giorgio et al., 2013). The detailed mechanism through which this transition is achieved are now the subject of intense study on a number of levels and will refocus the search for pharmacological modifier of the activity of the PTP both in MS and the many other pathological conditions in which the PTP plays a significant role.

## Conclusion

In 1868, Dr. Jean-Martin Charcot described his examination of a young woman with a particular tremor he had never seen before. He noted that she had other apparent neurological problems including slurred speech and abnormal eye movements. After her death, a brain autopsy was performed, and plaques or scleroses were noted upon examination. Charcot termed the disease “sclerose en plaques,” and this was the first clinical description of MS (Lublin, 2005). We have come a long way in our understanding of MS since Charcot first penned his description of the disease nearly 150 years ago. We now know that there is an immunologic component that leads to destruction of myelin by activated immune cells and ensuing manifestation of characteristic MS symptoms. This understanding has led to an extensive effort to develop immunomodulatory and immunosuppressant drugs to treat MS, which all work at some level to combat the pernicious autoimmune attack on the CNS, prevent the breakdown of the protective blood brain barrier, and suppress the infiltration of activated immune cells targeting myelin. Unfortunately, these drugs, while more or less adequate in treating relapsing-remitting MS, are unable to treat the progressive, debilitating decline in function that plagues a significant number of patients. Charcot, himself, noted neuronal loss in demyelinated lesions of MS patients, and this pathology has been convincingly confirmed with the aid of today's impressive technological tools.

While the study of neurodegenerative pathways in MS is still a fairly young area of research, considerable effort has been made in the past decade to understand the mechanisms involved and to define potential pharmacologic targets. Here, we have summarized some of the extensive work done investigating the effects of mitochondrial dysfunction on neurodegenerative processes in MS, with particular emphasis on the PTP, its modulator CyPD, and mitochondrial ROS sensor and amplifier p66. Thus, MS is not a mitochondrial disease, such as certain inherited mitochondrial diseases (e.g., Leber's optic neuropathy) but we believe mitochondrial dysfunction is a critical component of axonal injury within the acute focal inflammatory lesions and in the progressive neurodegenerative phase of the illness. It is the hope that with continued progress in these particular fields of research, multifaceted mitochondria-targeted neuroprotective therapies may soon be part of standard treatment regimen for not only MS but other neurodegenerative diseases as well.

### Conflict of interest statement

The authors declare that the research was conducted in the absence of any commercial or financial relationships that could be construed as a potential conflict of interest.

## Key Concept

Multiple sclerosis: Multiple sclerosis (MS) is one of the most common neurological diseases afflicting more than 2.1 million people worldwide. Of unknown etiology, classically MS has been considered an inflammatory disease in which both cellular and soluble mediators of inflammation result in multifocal demyelination. Thus, much effort has gone into developing therapies to control the pernicious immune response in MS. Over the past decade, however, the importance of axonal injury in MS has gained increasing attention and is now recognized as critical to the irreversible disability that occurs in this disease.
Neurodegeneration: Neurodegeneration is the umbrella term for the progressive loss of structure or function of neurons, including death of neurons and breakage or severing of axons. Many neurodegenerative diseases including Parkinson's, Alzheimer's, Amyotrophic lateral sclerosis, Huntington's and more recently MS occur as a result of neurodegenerative processes. As research progresses, many similarities appear which relate these diseases to one another on a sub-cellular level and in all cases, mitochondrial dysfunction has been proposed to play a critical role.
Mitochondrial dysfunction: The most common form of cell death in neurodegeneration is through various mitochondrial pathways. These pathways can control the activation of caspase-9 by regulating the release of cytochrome c from the mitochondrial intermembrane space (IMS). In addition, reactive oxygen species (ROS) are normal byproducts of mitochondrial respiratory chain activity. Over production of ROS (oxidative stress) is a central feature of all neurodegenerative disorders. Mitochondria are also involved with life-sustaining functions including calcium homeostasis, PCD, mitochondrial fission and fusion, lipid concentration of the mitochondrial membranes, and the mitochondrial permeability transition. Mitochondrial disease leading to neurodegeneration is likely, at least on some level, to involve all of these functions.
Reactive oxygen species: Reactive oxygen species (ROS) are potent intracellular oxidants that include hydrogen peroxide, superoxide, and hydroxyl radicals. They are commonly produced by dedicated enzymes, the NADPH oxidases, and as metabolic byproducts due to electron leaks by the electron transport chain. Because of their potential damaging effects on cellular macromolecules such as nucleic acids, proteins, and lipids, ROS levels are tightly regulated by intracellular superoxide dismutases and catalases. However, various stressors can dramatically increase ROS levels leading to significant intracellular damage. This is especially true for mitochondria, which are major sites of ROS production due to aerobic respiration and oxidative phosphorylation. ROS are potent activators of the mitochondrial permeability transition.
Mitochondrial permeability transition pore: The ability of mitochondria to take up and release calcium (Ca^2+^) is well established and the response of mitochondria to the addition of Ca^2+^ depends on the amount of Ca^2+^ added and the level of matrix Ca^2+^. If sufficient Ca^2+^ is accumulated within the mitochondrial matrix, a large increase in permeability of the inner mitochondrial membrane is induced with the loss of solutes with a molecular weight less than 1.5 kDa. This phenomenon is termed the mitochondrial permeability transition pore (PTP). In an isolated mitochondria preparation, opening of the PTP leads to swelling of the mitochondria, which is commonly used as a measure of PTP opening. However, although mitochondrial swelling occurs with isolated mitochondria undergoing PTP, mitochondrial swelling does not appear to occur in situ because of the high cytosolic level of proteins and solutes. In addition to Ca^2+^, opening of the PTP is triggered by ROS. The PTP has been implicated as playing a key role in apoptotic and necrotic cell death.
p66ShcA: The ShcA gene encodes two mRNA species, one specific for p66 and one, by the use of alternate translation start sites, encoding the p46 and p52 isoforms. While all three isoforms share the same amino acid sequence at the C terminus, including phosphotyrosine-binding (PTB), Src homology 2 (SH2), and collagen homology (CH1) domains, only the p66 isoform has a unique N terminus that allows it to be serine phosphorylated and targeted to mitochondria. Consequently, in contrast to p46 and p52, p66 is not involved in the transmission of activation signals from receptor tyrosine kinases to Ras proteins. Rather, the p66 isoform gets serine phosphorylated in the cytoplasm and subsequently targets to the mitochondrial intermembrane space. There, it serves as a redox enzyme by oxidizing cytochrome c, and transferring electrons to reduce oxygen into superoxide and hydrogen peroxide This localized pool of mitochondrial ROS mediates PTP opening and cell death activation.
